# Polymer and Hybrid Optical Devices Manipulated by the Thermo-Optic Effect

**DOI:** 10.3390/polym15183721

**Published:** 2023-09-11

**Authors:** Yuqi Xie, Liguo Chen, Haojia Li, Yunji Yi

**Affiliations:** 1 College of New Materials and New Energies, Shenzhen Technology University, Shenzhen 518118, China; 202100304027@stumail.sztu.edu.cn; 2 College of Integrated Circuits and Optoelectronic Chips, Shenzhen Technology University, Shenzhen 518118, China; 202100303008@stumail.sztu.edu.cn (L.C.);

**Keywords:** thermo-optic effect, thermo-optic switches, thermo-optic variable optical attenuators, thermo-optic temperature sensors, polymers

## Abstract

The thermo-optic effect is a crucial driving mechanism for optical devices. The application of the thermo-optic effect in integrated photonics has received extensive investigation, with continuous progress in the performance and fabrication processes of thermo-optic devices. Due to the high thermo-optic coefficient, polymers have become an excellent candidate for the preparation of high-performance thermo-optic devices. Firstly, this review briefly introduces the principle of the thermo-optic effect and the materials commonly used. In the third section, a brief introduction to the waveguide structure of thermo-optic devices is provided. In addition, three kinds of thermo-optic devices based on polymers, including an optical switch, a variable optical attenuator, and a temperature sensor, are reviewed. In the fourth section, the typical fabrication processes for waveguide devices based on polymers are introduced. Finally, thermo-optic devices play important roles in various applications. Nevertheless, the large-scale integrated applications of polymer-based thermo-optic devices are still worth investigating. Therefore, we propose a future direction for the development of polymers.

## 1. Introduction

Photonic devices have become ideal candidates for a range of applications, including solid-state LiDAR [1], microwave photonics [2], optical quantum computing [3], and on-chip optical sensing [4]. In integrated photonic technology, micro-electro-mechanical systems (MEMSs), electro-optic effect, and thermo-optic effect are commonly used mechanisms for optical signal modulation and processing. Photonic devices based on MEMSs, such as phase shifters, offer the advantages of high efficiency and the absence of thermal crosstalk. However, the packaging and long-term stability of MEMS devices still require further exploration. Photonic devices based on the electro-optic effect exhibit the advantage of fast modulation speeds and find widespread applications in the field of communications. Nevertheless, the electro-optic effect mechanism still faces challenges, particularly in terms of optical loss. In this review, our focus lies on the application of the thermo-optic effect in photonic devices, and the thermo-optic effect holds certain advantages in integrated photonic devices. Firstly, thermo-optic devices demonstrate long-term stability and low loss, facilitating their integration. Additionally, thermo-optic effect devices may be made in a relatively simple manner, and a variety of materials are available.

One of the key components in thermo-optic PICs is the thermo-optic switch and the thermo-optic variable optical attenuator (VOA). The basic principles of the thermo-optic VOA and the thermo-optic switch are similar. In recent years, an increasing need for enhanced thermo-optic switch performance in a variety of application scenarios has spurred intensive research efforts. In addition, with the development of information technologies, such as the Internet of Things and artificial intelligence, sensing technology has received more and more attention. The on-chip temperature sensor based on the thermo-optic effect is very attractive due to its easy integration and good stability.

Polymers are one of the important material systems of thermo-optic devices. The first important advantage of polymers is the large thermo-optic coefficient, which has been explored fruitfully in the realization of thermo-optic devices. Thermo-optical devices prepared by polymers can achieve a higher modulation efficiency. In addition, polymers allow for the easy and inexpensive preparation of optical waveguides. Due to the flexibility of polymers, the preparation processes for polymer optical devices are diverse. In terms of applications, polymers can regulate the properties of materials by doping, chemical modification, and other methods to meet different needs. It has the potential to deal with challenges such as high integration and low loss. The tunability of the properties of the polymer also expands the scope of application and provides more possibilities for the future development of polymer thermo-optic devices.

In this review, we mainly introduce three optical devices manipulated by the thermo-optic effect, including optical switches, VOAs, and optical waveguide temperature sensors. The fundamental principles underlying the thermo-optic effect are initially presented. Furthermore, relevant material parameters for commonly used materials in thermo-optic devices are provided. By taking advantage of the high thermo-optic coefficient of the polymer, the thermo-optic switch and the thermo-optic VOAs can achieve the function with low power consumption, and the thermo-optic temperature sensor can achieve high-sensitivity sensing. In the third section, following the introduction to the basic waveguide structure of thermo-optic devices, the research progress of polymer-based thermo-optic devices is reviewed. For low power consumption and high speed, we discuss the recent advancements in polymer thermo-optic switches, primarily focusing on materials, structures, and electrodes. At the same time, some progress in enhancing the sensitivity of thermo-optic temperature sensors is summarized. In the fourth section, the various fabrication processes for polymer waveguides are introduced and discussed in detail. Subsequently, Section 5 introduces the application of thermo-optic devices. We also highlight the challenges of polymer thermo-optic devices in practical applications and propose prospects based on the existing research. All in all, this review aims to provide comprehensive information about the thermo-optic devices of polymers and provide a broad overview for new researchers entering this field.

## 2. Principle of the Thermo-Optic Effect

The thermo-optic effect is one of the fundamental principles that enables the functionality of waveguide-type thermo-optical devices. This effect refers to changes in the refractive index of a material due to changes in temperature [5]. The thermo-optical coefficient, which is the temperature coefficient of the refractive index ($\frac{dn}{dT}$), is used to describe the rate at which the refractive index of a medium changes with temperature. T represents the temperature of the medium material, and n represents its refractive index. The formula for the thermo-optical coefficient can be expressed as [6]:

(1) $$\frac{dn}{dT}={\left(\frac{\partial n}{\partial \rho }\right)}_{T}\left(\frac{\partial \rho }{\partial T}\right)+{\left(\frac{\partial n}{\partial T}\right)}_{\rho },$$

or

(2) $$\frac{dn}{dT}=-{\left(\frac{\rho \partial n}{\partial \rho }\right)}_{T}\gamma +{\left(\frac{\partial n}{\partial T}\right)}_{\rho },$$

where ρ is the density and γ represents the thermal expansion coefficient. ${\left(\frac{\rho \partial n}{\partial \rho }\right)}_{T}\gamma$ represents the change in the refractive index caused by variations in the density of the medium. ${\left(\frac{\partial n}{\partial T}\right)}_{\rho }$ represents the change in the polarizability caused by variations in temperature while maintaining a constant density. To account for the change in the density of the material due to thermal stresses induced by temperature changes, the strain constant $\Lambda_{0}$ is introduced in the Lorentz–Lorenz (L–L) equation, which can be used to obtain:

(3) $${\left(\frac{\rho \partial n}{\partial \rho }\right)}_{T}=\left(1-\Lambda_{0}\right)\frac{\left(n^{2}-1\right)\left(n^{2}+1\right)}{6n}.$$

The $\Lambda_{0}$ value of the polymer is 0.1~0.2, indicating that the value of ${\left(\frac{\rho \partial n}{\partial \rho }\right)}_{T}$ is positive. The γ value of the polymer is approximately 2 × 10^−4^ K^−1^. In comparison, the thermal variation of the polymer’s refractive index is small (~−10^−6^ K^−1^). Therefore, the thermo-optic coefficient of polymers can be expressed as $-{\left(\frac{\rho \partial n}{\partial \rho }\right)}_{T}\gamma$, which is negative. For inorganic materials, the thermal expansion coefficient is relatively small. For example, the thermal expansion coefficient of fused quartz is approximately 10^−6^ K^−1^. Therefore, the thermo-optical effects in inorganic materials primarily arise from changes in the polarizability caused by temperature variations, specifically the second term in Equation (2).

The thermal properties of materials have a significant impact on the performance of thermo-optical devices. Therefore, designing high-performance thermo-optical devices requires a thorough understanding of the thermal characteristics of materials. Table 1 provides an overview of the refractive indices and thermal properties of several common materials.

Due to the special chemical structure and molecular motion mode of the polymer, it is possible to achieve a high thermo-optic coefficient by rationally designing the molecular structure of the polymer. The thermo-optic coefficient of some polymer materials has been recorded to surpass −10^−3^ K^−1^, according to the literature in print to date [7,8]. It is worth noting that this thermo-optical coefficient is an order of magnitude higher than that of traditional materials, such as silicon. Although such polymer materials have not yet been applied to thermo-optic devices, this provides the possibility of achieving thermo-optic devices with ultra-high modulation efficiency.

## 3. Thermo-Optic Devices

In recent years, thermo-optic devices based on polymer materials have made significant progress. In this section, we first introduce the common structures of thermo-optic devices. In addition, relevant research advances in the improvement of the performance of thermo-optical devices using polymeric materials are discussed and reviewed, including thermo-optical switches, thermo-optical VOAs, and thermo-optical temperature sensors.

**Figure 1:**
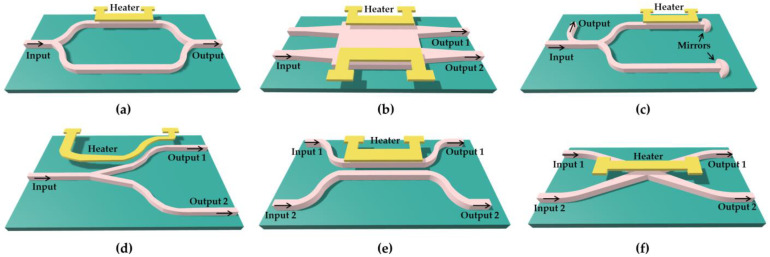
Various structures of thermo-optic devices. (**a**) Device with MZI, (**b**) device with MMI, (**c**) device with MI, (**d**) device with Y-branch, (**e**) device with directional coupler, and (**f**) device based on TIR.

The thermo-optic effect is the fundamental physical mechanism that enables the modulation of the refractive index in a material through temperature changes, thus achieving the operation of the thermo-optic switch. The thermo-optic effect confers several benefits for the thermo-optic switch, including low loss, facile fabrication, and compatibility with various materials and fabrication processes, endowing the thermo-optic switch with an appealing potential for applications in data communications, optical computing, and optical signal processing [9,10,11,12]. The thermo-optic temperature sensor also achieves the induction temperature change through this principle. Thermo-optic devices can be realized by interference-type Mach–Zehnder interferometers (MZIs) [13,14], multimode interference (MMI) [15,16], Michelson interferometers (MIs) [17,18], adiabatic evolution-type Y-branches [19,20], directional coupling (DC) [21,22], and total internal reflection (TIR) [23,24], as shown in Figure 1.

### 3.1. Thermo-Optic Switches and Thermo-Optic Variable Optical Attenuators

Polymer-based thermo-optic switches and thermo-optic VOAs possess the advantage of low power consumption. To further reduce the power consumption of polymer-based thermo-optic switches and thermo-optic VOAs while improving the response speed, this research has received considerable attention in recent years. Improving the performance of thermo-optical devices can be investigated from various aspects, including waveguide materials, waveguide structures, and electrode types. To achieve low-power and fast-response thermo-optical switches and thermo-optic VOAs, the use of polymers or hybrid integration is an effective approach. In addition, structural improvements or electrode optimization can further improve the performance of polymeric thermo-optical devices. In this section, related work to further reduce power consumption and time is presented. Finally, polymer-based thermo-optic switches and thermo-optic VOAs are compared with devices based on inorganic materials.

**Figure 2:**
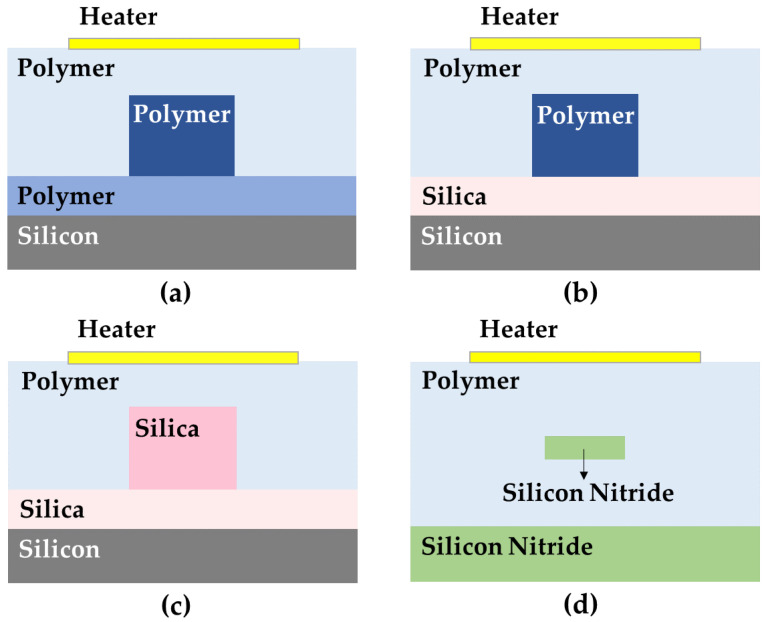
(**a**) All-polymer structure, (**b**) the structure of silica as the lower cladding layer and polymer as the core layer, (**c**) the structure of polymer as the upper cladding layer and silica as the core layer, and (**d**) the structure of polymer as the upper cladding layer and silicon nitride as the core layer.

In terms of materials, with a thermo-optical coefficient of ~−10^−4^ K^−1^ and low thermal conductivity, optical polymer materials are a desirable choice for low-power thermo-optic devices. Its use is currently the subject of extensive research and rapid development [25,26,27,28]. The cross-section of the all-polymer thermo-optic switch is shown in Figure 2a. Xibin Wang et al. proposed a low-power 1 × 2 polymer thermo-optic switch that can operate in the 650 nm short-distance communication window [29]. The team used the advantage of an adjustable refractive index in polymer synthesis to synthesize a stable cross-linkable polymer P(MMA-GMA) by the copolymerization of methylmethacrylate (MMA) and glycidyl methacrylate (GMA). The spectral absorption test indicated that the P(MMA-GMA) material system has a low-loss window of approximately 650 nm. The extinction ratio of the device at a 650 nm signal wavelength was greater than 23.4 dB, with a power consumption of only 5.3 mW. The rise time and fall time of the device were 464.4 µs and 448.0 µs, respectively. Whereas inorganic materials have a large thermal conductivity, polymers have a large thermo-optic coefficient. Consequently, organic/inorganic hybrid integrated devices can combine the advantages of both materials and produce thermo-optic switches with a low power consumption and fast response [30,31,32,33,34]. The thermo-optic switch cross-section of the hybrid integrated structure is shown in Figure 2b. Yunfei Yan et al. reported a thermo-optic switch that employed polymer as the core layer and upper cladding layer and silica as the lower cladding layer [35]. By using silica instead of polymer as the lower cladding layer, the switching time of the device was reduced by 40% compared with the all-polymer structure of the thermo-optic switch. At a driving power of 7.2 mW, the rise time and fall time of the switch were 106 µs and 93 µs, respectively. Another hybrid integrated structure using an inorganic material as the core layer and a polymer as the cladding was also investigated. In 2022, a thermo-optic switch with silica as the core layer and polymer as the cladding was demonstrated [36]. A cross-sectional view of the switch is depicted in Figure 2c. The width of the core in the heating region was reduced so that the optical field is located more on the polymer cladding, enabling more efficient thermal tuning. The simulation results show that the power consumption of the hybrid integrated structure is about 95% lower than that of the all-silica structure. This structure retains the low-loss characteristics of silica while taking advantage of the large thermo-optical coefficient of the polymer to reduce power consumption and is expected to be used in optical communications. Recently, the silicon nitride platform has gained significant attention due to its low transmission loss from visible to mid-infrared wavelengths, compatibility with CMOS processes, and high refractive index [37,38,39,40]. Although the thermo-optic coefficient of silicon nitride is not high, it has a good compatibility with polymeric materials. The power consumption of the thermo-optic switch using silicon nitride waveguides can be reduced by taking advantage of the large thermo-optic coefficient of polymers and the simplicity of fabrication. In 2023, Xinhong Jiang et al. proposed a thermo-optic switch with a thin silicon nitride waveguide embedded in a polymer cladding [41]. The material distribution is shown in Figure 2d. Since a large portion of the optical field is located on the polymer cladding, the waveguide can be thermally tuned efficiently.

**Figure 3:**
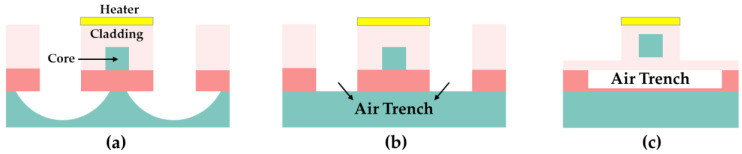
(**a**) The structure with suspended arms, (**b**) the structure with air trenches, and (**c**) the structure with a bottom air trench.

In terms of the waveguide structure, air trenches are often introduced to prevent heat diffusion for the purpose of reducing power consumption [42,43]. The thermal conductivity of air at room temperature is ~0.025 W/mK, whereas that of polymers is 0.1–0.3 W/mK. Consequently, the most common approach is to leverage the low thermal conductivity of air by etching the air trench at the bottom or side of the heating arm, as shown in Figure 3. In 2017, Sun Shiqi et al. etched air trenches on both sides of the waveguide core [44]. The power consumption of the thermo-optic VOAs was reduced from 8.71 mW to 2.80 mW. In 2022, Long Zhang et al. discussed the effect of the device structure on the core layer temperature by simulating the temperature field of the heated waveguide [45]. The data showed that the presence of bilateral air trenches increased the core layer temperature of conventional rectangular waveguides by about 52.6%. In 2021, Kai Chen et al. used the structure of a laterally supported suspended ridge waveguide (LSSRW) combined with a bottom air trench for a thermo-optic switch [46]. This suspended structure is also applicable to polymer-based thermo-optic devices, but structural stability issues need to be considered.

**Figure 4:**
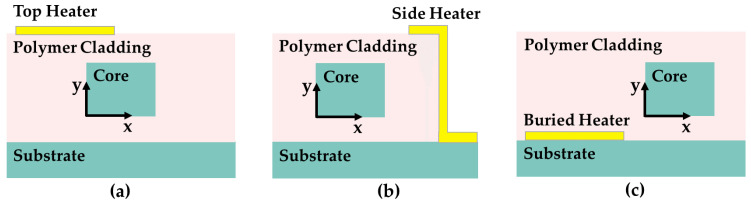
Cross sections of the waveguides with (**a**) top heater, (**b**) side heater, and (**c**) buried heater.

**Figure 5:**
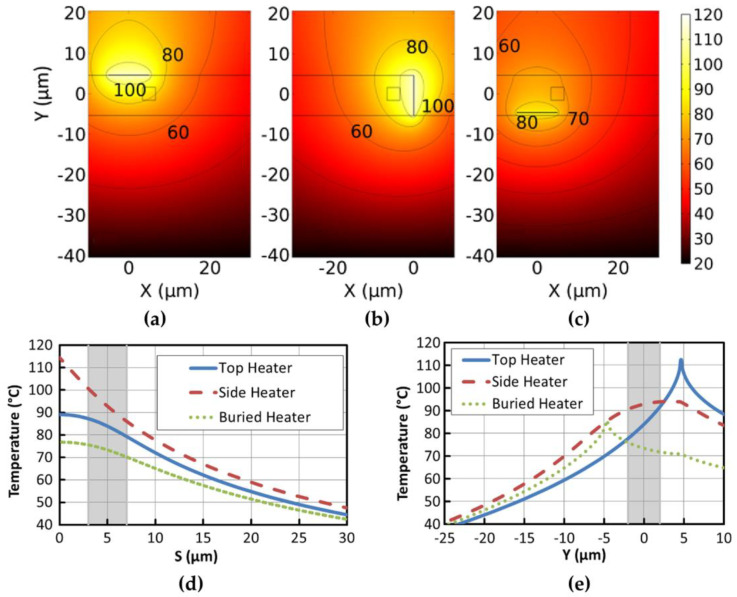
Cross sections of the thermal distribution of the waveguides with (**a**) the top heater, (**b**) the side heater, and (**c**) the buried heater. (**d**) Comparisons of the temperature gradients along the X-axis in the waveguide center. (**e**) Comparisons of the temperature gradients along the Y-axis in the waveguide center. Reprinted with permission from [47]. © Optical Society of America (2015).

As part of a thermo-optic switch, it is necessary to optimize the heater to further improve the heating efficiency. One aspect of optimization involves the layout of the electrode. Traditional heating schemes for thermo-optical devices were mostly limited to top electrodes. Alejandro Maese-Novo et al. conducted a series of thermal-optic simulations to determine the optimal heating schemes [47]. As depicted in Figure 4, the heater electrodes can be structured on the top of the polymer cladding (top heater), buried underneath the waveguide (buried heater), or deposited on the sidewall of the air trench (side heater). Figure 5a–c show the temperature distributions. Horizontally oriented planar waveguide thermo-optic devices require a temperature gradient to be applied across the waveguide to function. Therefore, the positioning of the top and bottom heaters is shifted away from the center of the waveguide. The ambient temperature is set to 20 °C, and the temperature gradient distribution curves in the X and Y directions for the three layouts are provided in Figure 5d,e. In the shaded area, which represents the waveguide region, it has been found that the side electrode is most efficient at producing a temperature gradient in the horizontal direction without disturbing the uniform temperature distribution in the vertical direction. Two types of VOAs with different structures were manufactured with the best-performing side heater. The MMI-based VOA provides a 37 dB attenuation range at a power of 10.1 mW, whereas the VOA based on MZI exhibits an attenuation range of 51 dB at a significantly lower power of 1.8 mW for heater operation.

Another aspect of optimization pertains to electrode materials. Metal is typically utilized for the electrode heater in thermo-optic systems. However, metal layering can result in light absorption, thereby necessitating a buffer layer with substantial thickness between the waveguide core and the electrode. Unfortunately, this buffer layer may restrict and hinder the heating efficiency of the system. In recent years, two-dimensional layered materials have drawn increasing amounts of interest in electronics and photonics due to their special physical properties [48,49,50,51]. For example, graphene can be used as a thermal diffuser and flexible heater because of its high thermal conductivity (5300 W/(mK)) [52,53,54]. Especially in polymer devices, graphene electrodes can be buried by using the advantages of the easy processing of polymers [55,56,57]. In 2019, taking advantage of the low absorption loss of graphene to transverse magnetic (TM) waves, Xibin Wang et al. used graphene as an electrode to be directly buried at the bottom of the waveguide without needing any buffer layer [58]. According to the test results, the switching power using a graphene electrode (~2.1 mW) was nearly four times lower than that obtained with an Al electrode (~7.8 mW), and graphene electrodes also decreased the response time. However, the polarization dependence of the device is strong, which is not conducive to the application of the device. In 2020, a thermo-optic switch based on a graphene electrode with negligible light absorption for both transverse electric (TE) and transverse magnetic (TM) polarizations was manufactured [59]. By using a 6 μm × 6 μm square core, the TE and TM polarized optical fields were constrained, which resulted in nearly little overlap with the top graphene. Therefore, for both TE and TM, graphene only produced a negligible light absorption. According to the simulation results, the loss of TE and TM polarization was only 0.0995 dB/mm and 0.0694 dB/mm, respectively.

**Figure 6:**
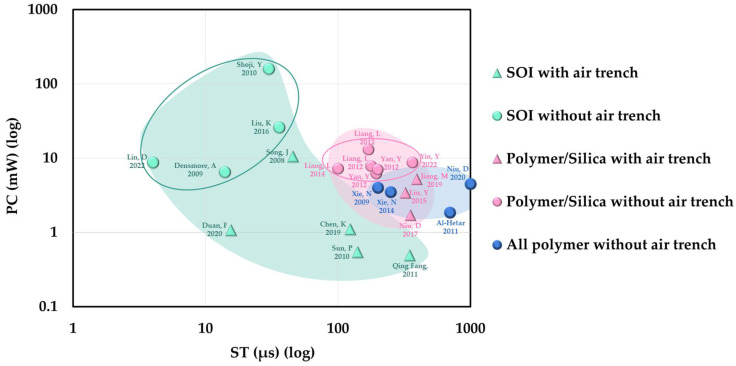
Performance of various thermo-optic switches regarding power consumption and switching time. The power consumption and switching time are indicated for switches using SOI with air trench [62,63,64,65], SOI without air trench [18,46,60,61,67], polymer/silica with air trench [31,33,42], polymer/silica without air trench [35,76,77,78,79,80], all polymer without air trench [71,72,73,74].

Compared with thermo-optic switches and thermo-optic VOAs based on other materials, it is helpful to understand the characteristics and advantages of polymer materials more comprehensively. Some typical thermo-optic switches and thermo-optic VOAs are organized in Table 2. Figure 6 demonstrates the performance of multiple thermo-optic switches in two dimensions (power consumption and response time) simultaneously.

**Table 2:** Performance comparison of typical thermo-optic switches.

Material	Structure	Heater	PC (mW) ^1^	ST (µs) ^2^	Reference
SOI ^3^	DC-MZI with suspended phase arms	TiN	0.49	348	[60]
	MZI with free-standing waveguides	Pt	0.54	141	[61]
	MZI with densely folded waveguides	Cr and Au	6.5	14	[62]
	MZI with spiral waveguides	TiN	8.73	4	[63]
	MI with air trenches	Titanium	10.5	45.8	[18]
	DC-MZI	Pt	160	30	[64]
	Rectangular MZI	Al	26	36	[65]
	MRR ^4^	Graphene	14.42	7.68	[66]
	MZI with LSSRW	Cr and Cu	1.1	124	[67]
	MZI with LSSRW	Cr and Au	1.07	15.6	[46]
Silica on silicon	DC-MZI with a suspended narrow ridge structure	-	20	-	[68]
	DC-MZI with air trenches	Ti and Wu	155	-	[69]
	MZI with air trenches	Titanium	95	-	[70]
Polymer	MMI-MZI	Cr and Al	1.85	700	[71]
	MZI	Al	4.5	1000	[72]
	MMI-MZI	Ni and Ti	<4	200	[73]
	MMI-MZI	Ti and Ni	3.5	250	[74]
	DC-MZI	Graphene	3.30/3.12	1500	[59]
	MZI	Graphene	1.57	71.8	[75]
Polymer/Silica	DC-MZI	Al	7.2	100	[76]
	MZI	Al	13	170	[77]
	MZI	Al	7.8	178	[78]
	DC-MZI	Al	6.2	194	[79]
	DC-MZI	Al	<7.2	199	[35]
	MMI-MZI	Al	8.72	364	[80]
	MZI with air trenches	Al	5.2	393.3	[31]
	MZI with air trenches	Al	1.7	353.2	[33]
	MZI with air trenches	Al	3.4	323	[42]

^1^ PC, power consumption; ^2^ ST, switching time; ^3^ SOI, silicon-on-insulator, ^4^ MRR, microring resonators.

The SOI platform is highly attractive due to its potential for integrating optical components onto the same substrate. Additionally, based on the high thermo-optic coefficient and thermal conductivity of the material, as well as the more compact device structures, thermo-optic devices on the SOI platform exhibit excellent performance. However, in certain specific application scenarios, polymer devices are capable of meeting the requirements and may even possess advantages. Firstly, polymers exhibit a high thermo-optic coefficient and have the potential for further improvement. The low thermal conductivity characteristic of polymers helps in reducing power consumption in thermo-optic devices. However, it can be unfavorable for response speed. Therefore, in scenarios where the emphasis is on minimizing power consumption with a lower requirement for response speed, thermo-optic devices based on polymer materials are suitable candidates. Secondly, in terms of device packaging, polymer devices offer simplicity and cost-effectiveness. Due to the low refractive index contrast of polymers, the dimensions of polymer waveguides can be matched with those of optical fibers, allowing for direct coupling between them. Thirdly, in terms of device fabrication, polymer devices offer a simplified and cost-effective manufacturing process. Polymer waveguides can be easily fabricated at room temperature using techniques such as spin coating and UV lithography. Additionally, novel large-scale fabrication methods, such as nanoimprinting, have significantly enhanced the industrialization potential of polymer photonic devices. Moreover, due to the flexibility in the fabrication of polymer devices, achieving three-dimensional integration is easier, thereby improving the overall integration density. Lastly, in terms of applications, the flexible nature of polymers, allowing for bending, folding, and stretching, provides polymer devices with unparalleled advantages in the field of flexible photonics technology. Compared to traditional materials-based thermo-optic devices, polymer thermo-optic devices have a unique application potential in areas such as wearable sensors, flexible optical interconnect chips, and virtual reality/augmented reality (VR/AR) technologies. The ability of polymer devices to conform to curved surfaces and accommodate varied form factors makes them highly suitable for emerging applications requiring flexibility and adaptability.

For polymer thermo-optic switches, it is a common optimization method to reduce the driving power consumption and thermal crosstalk by using air to prevent heat diffusion. However, the response time may be adversely affected. Taking advantage of the easy-doping characteristics of polymers, researchers have proposed that doping graphene in polymers can help to improve the response speed of thermo-optic switches [81]. Furthermore, the type of heater utilized is also a significant factor to consider. From the perspective of heat diffusion, a narrower top electrode is advantageous for reducing the driving power. Nevertheless, it poses a significant challenge to machining accuracy. From the perspective of the heater layout, the side heater can offer a horizontal temperature gradient for planar optical waveguide devices to achieve effective heating. The optimization of the heater layout could be further improved by modifying the dimension of the waveguide core, adjusting the substrate thickness, and altering the distance between the electrode and the core. However, the implementation of these optimization measures may result in adverse effects on the response speed. In conclusion, the trade-off between driving power and response time is a crucial consideration for researchers during the design and manufacturing of thermo-optic switches. In recent years, with the assistance of 2D materials and other materials, thermo-optic switches have made breakthroughs in power consumption and response speed [75,82,83]. The compactness and mechanical flexibility of 2D materials can also meet the requirements of high integration. On the other hand, the 2D material microheater is faced with the problem of complex preparation and transfer processes. Inspired by the related research on silicon-based thermo-optic switches, hydrogen-doped indium oxide (IHO) microheaters with low loss and high-efficiency preparation may become a new solution [84].

### 3.2. Thermo-Optic Temperature Sensors

The thermo-optic effect can be utilized in the thermo-optic temperature sensor, wherein variations in temperature are detected by monitoring alterations in the propagation of light through a material with a temperature-dependent refractive index. Thermo-optic temperature sensors based on polymers have the advantages of high sensitivity and a wide application range, which can be used in various applications, including bioengineering, analytical chemistry, environmental monitoring, and food preservation. In this section, we focus on sensing sensitivity and discuss related research on polymer thermo-optic temperature sensors.

**Figure 7:**
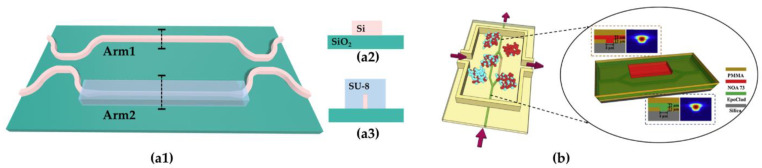
(**a1**) A diagram of the MZI-based temperature sensor with silicon/SU-8 hybrid waveguides. The cross sections of (**a2**) arm1 and (**a3**) arm2. (**b**) A schematic diagram of a temperature sensor integrated into a microfluidic system (reprinted from [86], with permission from Elsevier).

In terms of materials, the thermo-optic coefficient of the waveguide cladding material has an important influence on the sensitivity of the temperature sensor. In 2016, Xiaowei Guan et al. developed a temperature sensor based on MZI using an SU-8 cladding layer on the outer side of one narrowed waveguide arm, as shown in Figure 7(a1,a2,a3) [85]. Because SU-8 has a negative TOC (−1.21 × 10^−4^ K^−1^), the two arms consist of hybrid waveguides that induce opposite phase changes. The two arms consist of hybrid waveguides that provide opposite phase changes and increased sensitivity to 172 pm/°C. In 2019, Donghai Niu et al. employed NOA73 with a thermo-optic coefficient of ~3 × 10^−4^ K^−1^ and EpoClad with a thermo-optic coefficient of −1.18 × 10^−4^ K^−1^ as the core of the two waveguide arms of an MZI, achieving a sensing accuracy of 1.685 °C^−1^ [86]. Figure 7b depicts the structure of the temperature sensor, which utilized a silica lower-cladding layer to improve the response time. This sensor is oriented toward micro-fluidic system applications and can accurately measure the temperature of biological growth environments in real time by taking advantage of the biocompatibility of the polymer material.

**Figure 8:**
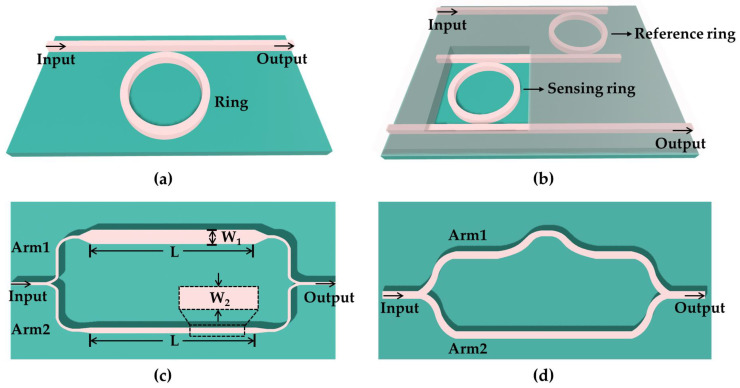
(**a**) A three-dimensional diagram of an MRR structure. (**b**) A three-dimensional diagram of the CRR temperature sensor. (**c**) A schematic of the asymmetric MZI temperature sensor with two waveguide arms of different widths. (**d**) A schematic of the asymmetric MZI temperature sensor with two waveguide arms of different lengths.

In terms of structures, MZI and MRR are the two fundamental structures of planar optical waveguide temperature sensors. The microring structure is shown in Figure 8a, which has the advantages of miniaturization and high sensitivity [87,88]. In 2010, Gun-Duk Kim et al. used silicon to create an ultrasmall photonic temperature sensor with an MRR structure [89]. The best sensitivity obtained was 83 pm/°C when the waveguide width was 500 nm, which is typically the sensitivity limit of a single MRR-type temperature sensor based on all silicon. Therefore, researchers have improved the sensitivity of temperature sensors by using special structures. Among these, the cascaded ring resonator (CRR) technique is a popular method for increasing the sensitivity of optical sensors through the generation of the Vernier effect [90,91,92]. The device structure is shown in Figure 8b. One of the two ring resonators is used as a reference ring to generate a Vernier effect, and the other is used as a detecting ring to interact with the external medium as a sensing resonator [93]. The reference resonator is preferably temperature-insensitive. Therefore, the traditional CRR sensor adopts a multimask manufacturing process to achieve the thermal isolation of the reference ring, which increases the process cost and complexity. In 2016, Hyun-Tae Kim et al. used two ring resonators with distinct temperature sensitivities and free spectral ranges (FSRs) as sensors [94]. The proposed method eliminates the necessity of isolating one of the ring resonators and facilitates the implementation of the sensor via a single-mask CMOS-compatible process. The experimental results indicated that the temperature sensitivity is 293.9 pm/°C, which is 6.3 times higher than that of a single MRR. The Vernier effect configuration includes CRR, two cascaded MZIs (CMZI), a cascaded MRR, and MZI [95,96,97]. The above design ideas can be applied to polymer-based thermo-optic temperature sensors. Increasing the refractive index of the polymer to reduce the radius of the microring is a problem that needs to be solved in this process. For temperature sensors based on MZI, achieving a high sensitivity by modulating the group refractive index in two-beam interference is another low-cost manufacturing method [98]. In 2018, Yang Zhang et al. fabricated an MZI-based temperature sensor on the SOI platform, achieving an enhanced sensitivity exceeding 438 pm/°C [99]. The two arms of the MZI are equal in length but different in width, as shown in Figure 8c. The temperature sensitivity can be obtained by Equation (4) [99]:

(4) $$\frac{\partial \lambda }{\partial T}=\frac{\lambda }{\left|n_{g}\left(W_{1}\right)-n_{g}\left(W_{2}\right)\right|}\cdot \left[\frac{\partial n_{eff}\left(W_{1}\right)}{\partial T}-\frac{\partial n_{eff}\left(W_{2}\right)}{\partial T}\right],$$

where λ is the wavelength and $n_{eff}\left(W_{1}\right)$ and $n_{eff}\left(W_{2}\right)$ are the effective indices of the waveguide with widths of $W_{1}$ and $W_{2}$, respectively. $n_{g}\left(W_{i}\right)=n_{eff}\left(W_{i}\right)-\lambda \frac{\partial n_{eff}\left(W_{i}\right)}{\partial \lambda }\left(i=1,2\right)$ represents the group index of the fundamental mode with waveguide width $W_{i}$. The equation illustrates that the temperature sensitivity is influenced by the disparity in the group index difference (GID) between the waveguides in the two MZI arms as well as the variation in the temperature sensitivities of their effective mode refractive indices. Hence, to achieve high sensitivity, the widths of the two waveguides are adjusted to have disparate temperature sensitivities while maintaining nearly identical group indices. In 2019, an all-polymer temperature sensor based on the principle of two-beam interference was proposed [100]. The sensitivity of the sensor was measured to be 22.7 nm/°C by using NOA73 with a large thermo-optic coefficient of ~−3 × 10^−4^ K^−1^. In addition, the sensor based on the MZI structure can also use the asymmetric length of the interference arm to achieve sensing, as shown in Figure 8d. The temperature sensitivity increases with the increase in the phase difference between the two arms, that is, the sensitivity of the temperature sensor is positively correlated with the length difference between the two arms. When designing temperature sensors based on polymers, the balance between bending loss and sensitivity should be taken into account.

Furthermore, photonic temperature sensors can also adopt the structure of waveguide Bragg gratings (WBGs). Changes in the grating period and refractive index induced by temperature variations lead to a drift in the central wavelength of the grating, enabling the sensor to detect changes in temperature. Compared to fiber Bragg gratings (FBGs), WBGs offer advantages, such as multifunctional integration, stability, and a diverse range of material choices. Polymers have attracted significant attention in this field because they are suitable for fabricating WBG-based thermo-optic temperature sensors with a high sensitivity and flexible process. In 2016, Nuria Teigell Benéitez et al. utilized imprinting to define the gratings and employed a new capillary filling-based replication method to fabricate waveguides [101]. This WBG-based temperature sensor was prepared using a hybrid inorganic–organic material (Ormocer^®^). It demonstrated a sensitivity of −249 pm/°C, which is 25 times higher than that of normal silica fiber. In 2018, Liang Tian et al. reported a polymer/silica hybrid WBG [102]. The Bragg grating was fabricated on a silica cladding using contact lithography and inductively coupled plasma etching, while the polymer waveguide was prepared through ultraviolet bleaching. The experimental results showed that the temperature sensor exhibited a high temperature sensitivity, with a peak shift of 1.5 nm with a temperature variation in the range of 25–35 °C.

Polymer-based temperature sensors were compared with sensors of other materials, and a summary is shown in Table 3. For an optical waveguide sensor, the sensitivity is determined by two main factors. The first factor is the waveguide sensitivity, which largely depends on the choice of optical waveguide material. For the thermo-optic temperature sensor, the sensitivity of the optical waveguide is related to the thermophysical properties of the material. As previously discussed, the choice of waveguide material needs to be carefully considered given that different material platforms possess distinct strengths and limitations. Polymers with large thermo-optic coefficients provide a great opportunity for thermo-optic temperature sensors to achieve a high sensitivity.

The second factor is the device sensitivity, which is determined by the design of the device. There are two popular types of structures. One is based on MRR, which is ultracompact but has a sensitivity limit. The other structure is asymmetric MZI, which is based on two-beam interference. For asymmetric MZI structures, three main methods are employed: two-arm length asymmetry, two-arm width asymmetry, and two-arm material asymmetry. Moreover, the Vernier effect also offers a method for enhancing sensitivity by cascading two resonators or interferometers.

## 4. Fabrication

Compared with inorganic optical waveguides, polymer optical waveguides have the significant advantages of simple processing and inexpensive preparation. In parallel with the boom in polymer optical devices, the fabrication processes of polymer waveguides have been widely studied. In this section, we focus on the preparation processes of polymer waveguides, which are also applicable to thermo-optic devices based on polymers. Additionally, the benefits and drawbacks of various processes were examined.

### 4.1. Photolithography

**Figure 9:**
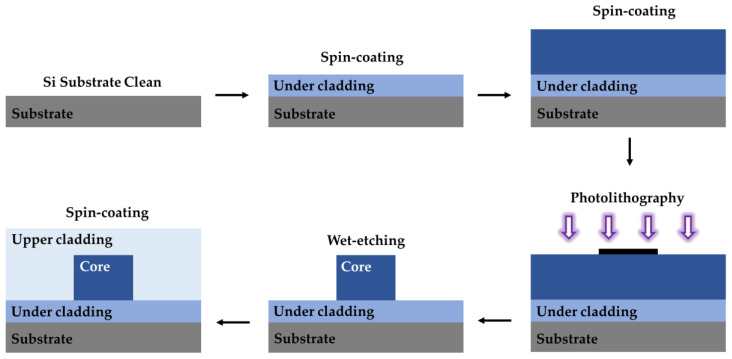
Fabrication process for polymer waveguides using wet etching. Purple arrows represent ultraviolet light.

**Figure 10:**
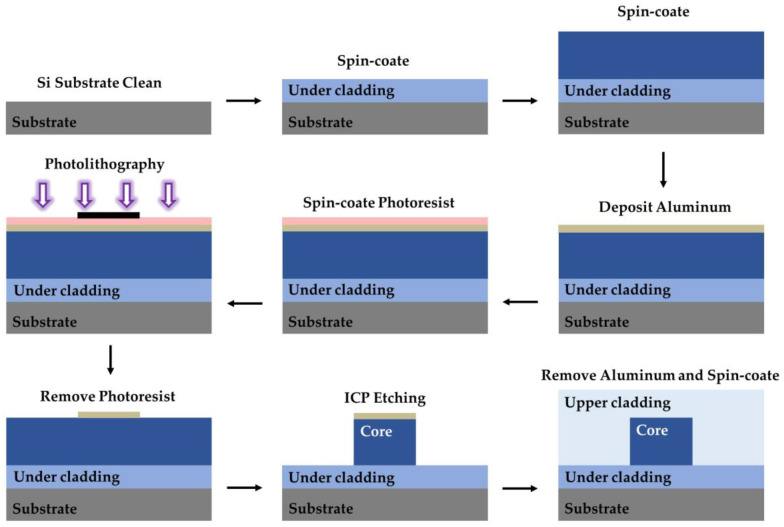
Fabrication process for polymer waveguides using ICP etching. Purple arrows represent ultraviolet light.

Photolithography with exposed masks is a traditional polymer waveguide fabrication process. The process steps combined with wet etching are shown in Figure 9. First, the polymer is spin-coated on the cleaned and dried substrate, and the lower cladding is formed after ultraviolet (UV) curing. Next, the core layer is spin-coated, UV photo-etched using a mask, and post-baked. Then, the developer is used to clean the graphics and fabricate the waveguide core. The upper cladding is fabricated on the core layer by spin-coating. Finally, the whole device is UV-cured or heated. Wet etching has the advantages of high production efficiency and simple equipment, but it also produces chemical liquid waste. The preparation of polymer devices can also be achieved by dry etching, including reactive ion etching (RIE) and inductively coupled plasma (ICP) etching. The device fabrication process using ICP etching is shown in Figure 10. Dry etching has the advantages of high controllability and no chemical waste. However, the cost of dry etching is high, and the equipment is complex.

### 4.2. Photobleaching

**Figure 11:**
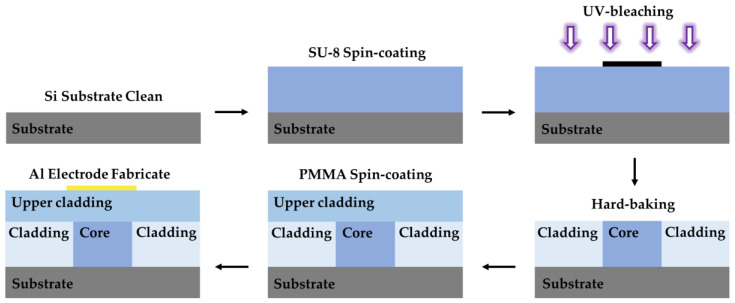
Fabrication process for polymer devices using photobleaching. Purple arrows represent ultraviolet light.

For polymers possessing photosensitive properties, photobleaching is a simple approach to adjusting the refractive index of the film and manufacturing optical waveguides. For instance, SU-8, which exhibits UV-sensitive characteristics, was investigated by researchers who discovered that, at a constant exposure degree, the refractive index of the SU-8 film is related to the post-baking temperature. Specifically, the refractive index of the exposed film diminishes with an increase in the post-baking temperature, while that of the unexposed film amplifies with the same parameter. As a result, the optical waveguide can be prepared by adjusting the exposure time and the post-baking temperature. In 2014, Xibin Wang et al. prepared a thermo-optic switch using a simple thermal UV bleaching technique, as shown in Figure 11 [109]. The process of preparing optical waveguides by photobleaching is simple. However, photobleaching does not produce large refractive index changes. The experiments showed that the refractive index of unexposed SU-8 crosslinked by thermal initiation is 0.0072 higher than that which is cross-linked via UV exposure and post-baking [110]. Consequently, the optical waveguide produced through photobleaching suffers from an insufficient refractive index difference and bending loss issues.

### 4.3. Nanoimprint

**Figure 12:**
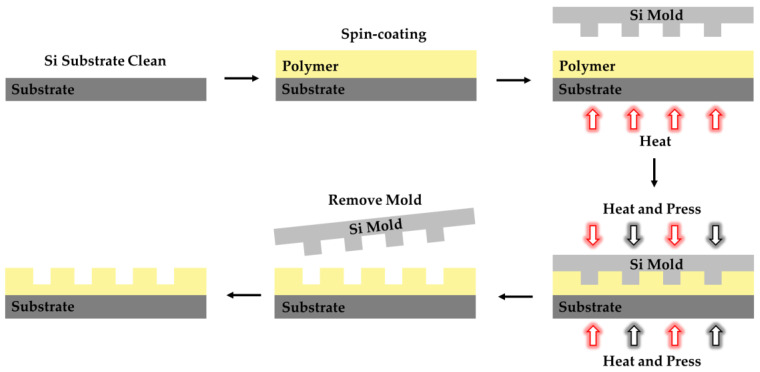
Fabrication process for polymer waveguides using T-NIL. The red arrow represents the direction of heating. The black arrow represents the direction of pressure.

**Figure 13:**
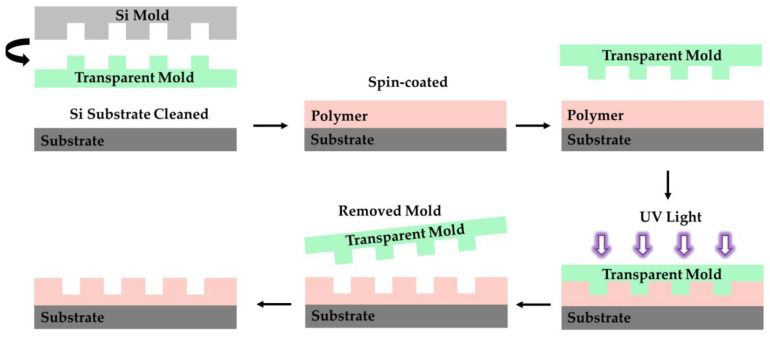
Fabrication process for polymer waveguides using UV-NIL. Purple arrows represent ultraviolet light.

Nanoimprint lithography (NIL) is divided into thermal nanoimprint lithography (T-NIL), ultraviolet nanoimprint lithography (UV-NIL), and microcontact printing (μCP). The process of preparing polymer optical waveguides by T-NIL is shown in Figure 12. First, the required nanopatterns are prepared on hard silicon wafers by electron beam lithography. Then, the polymer is spin-coated on the substrate and heated above the glass transition temperature (Tg). the mold is pressed into a softened polymer film and the polymer is solidified by cooling. After demolding, the pattern on the mold is transferred to the polymer film. Finally, follow-up processes are selected according to the requirements, such as spin-coating or etching the remaining resist layers. Unlike T-NIL, preparing optical waveguides based on UV-NIL can be conducted at room temperature. The fabrication process of optical waveguides by UV-NIL is shown in Figure 13. The most common transparent mold is polydimethylsiloxane (PDMS) due to its transparency to UV irradiation. Then, the transparent mold is pressed onto the polymer film. Under UV irradiation for a period of time, the polymer is cured by a crosslinking reaction. μCP requires a soft mold, and the nanoimprinting process is conducted by molecular self-assembly. Compared with the existing technology, NIL has the advantages of large-scale and low-cost preparation. The process is simple while ensuring high precision. However, the residual layer requires subsequent etching steps, which is an important problem that nanoimprinting faces at present.

### 4.4. Direct Laser Writing

**Figure 14:**
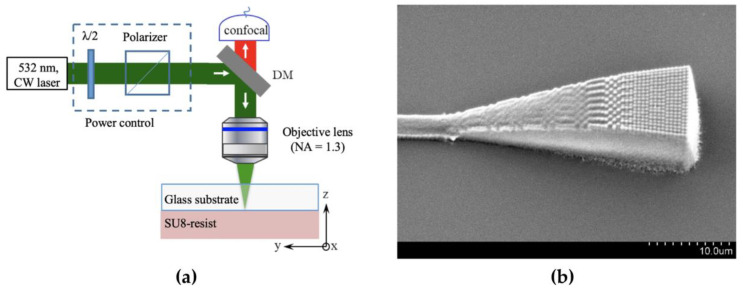
(**a**) The schematic diagram of LOPA-DLW (reprinted from [112], licensed under a Creative Common Attribution 4.0 Generic License. https://creativecommons.org/licenses/by/4.0/). (**b**) SEM of the tapered grating coupler (reprinted from [112], licensed under a Creative Common Attribution 4.0 Generic License. https://creativecommons.org/licenses/by/4.0/).

The preparation of micro–nano structures by direct laser writing (DLW) technology can be divided into photoablation and photopolymerization [111]. Photoablation refers to the process in which the polymer surface is melted and evaporated after being irradiated by a high-energy laser beam. Photopolymerization is a technology that solidifies the liquid, photosensitive polymer in the scanning path by laser irradiation. In 2023, Thuy Linh La et al. realized a tapered grating coupled structure with a gap of 500 nm by using low one-photon absorption (LOPA) DLW technology, as shown in Figure 14 [112]. The DLW technology has the advantages of high processing resolution, simple process, and no mask exposure. DLW technology based on two-photon absorption (TPA) can also be used to prepare 3D microstructures with high processing flexibility. However, the processing rate is an important reason for limiting the application of DLW technology. At the same time, the preparation results are affected by laser power, polymer type, and processing speed. DLW technology needs to ensure the consistency of parameters to have high repeatability.

### 4.5. Dispensing Direct Writing

**Figure 15:**
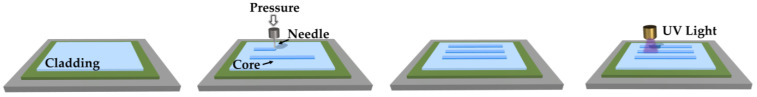
Fabrication process for polymer waveguides using dispensing direct writing technology.

Dispensing direct writing is a process that combines high-viscosity polymer materials and a high-precision liquid dispenser to prepare optical waveguides [113,114,115]. The details of preparing polymer waveguides using dispensing direct writing are shown in Figure 15. First, the cladding material is coated on the substrate, and the tip of a needle with the core material is inserted into the cladding. The 3D manipulator controls the needle to move according to the designed path, and the needle dispenses the material into the cladding material under pressure. Finally, the waveguide is cured by UV irradiation and then lifted off from the substrate. Before curing, the core material and cladding material are diffused. By controlling the waiting time before curing, the refractive index distribution and the cross-section shape of the waveguide core can be adjusted. On the other hand, due to the influence of liquid flow, the waveguide prepared by dispensing direct writing technology is disturbed by the cladding colloid. Therefore, the accuracy of the dispensing direct writing technology is limited, especially in the waveguide spacing.

## 5. Summary and Discussion

As the performance of thermo-optic elements continues to improve, they become attractive for more applications. For example, reconfigurable photonic integrated circuits (PICs) can perform various functions based on the same structure by densely composing thermo-optic elements, which have attracted great interest and flourished [116,117]. Thermo-optic devices with a high modulation efficiency pave the way for optical communication [118,119], quantum photonics [120,121], optical computing [10,122,123], microwave photonics [124,125], and optical phased array [126,127]. However, photonic devices are developing towards integration and industrial applications, and inorganic materials provide a good platform for this. Polymer thermo-optic devices have not yet achieved advantages comparable to inorganic materials and still face the following challenges.

First, the low refractive index (<1.70) is an obstacle to achieving the high-density integration and miniaturization of polymer photonic devices. Figuring out how to raise the polymer’s refractive index is one of the key issues. In recent years, high-refractive-index polymers (HRIPs) have been studied and reported [128,129,130]. Chemical synthesis is a common method to prepare high-refractive-index polymers [131]. Highly polarized and compact substituents in polymers, such as aromatic, sulfur, phosphorus, and selenium, have been introduced. Another method is doping nanoparticles with a high refractive index to form polymer nanocomposites [132,133]. The nanoparticles used as high-refractive-index dopants include TiO_2_ (refractive index = 2.5–2.7), ZrO_2_ (refractive index = 2.15–2.2), ZnS (refractive index = 2.36), and PbS (refractive index = 4.20). The development of high-refractive-index polymers is expected to expand the preparation of polymer photonic components to the level of large-scale integrated chips. Second, polymer materials may exhibit high optical losses in the optical frequency range. An essential difficulty is figuring out how to lower the optical loss of polymers, particularly in the context of data transfer, such as optical communication. Recently, halogenated polymers have been suggested as a solution to the issue of high transmission loss (>0.2 dB/cm in 1300–1650 nm) [134,135,136,137]. The low absorption loss of polymers in the 1300–1650 nm wavelength range is achieved by using fluorocarbon bonds (C-F) instead of hydrocarbon bonds (C-H). For example, the absorption loss of perfluoropolymer materials in the communication band is less than 0.01 dB/cm. The loss of polymer devices can be effectively reduced through a reasonable selection of low-loss polymers, together with the optical waveguide surface smoothing treatment technology. In addition, polymers have the property of being easily doped [138]. By doping the polymer with rare-earth ions, such as Yb^3+^, Tm^3+^, and Er^3+^, an excited radiation process can be introduced to achieve the amplification of the optical signal, which can offset the optical losses [139,140,141]. Third, low thermal conductivity is an important factor limiting the rapid response of all-polymer thermo-optic devices. However, a low thermal conductivity also makes polymer-based thermo-optical devices maintain the advantage of low power consumption. Therefore, with the help of other materials with high thermal conductivity, complementary advantages can be achieved. As already mentioned, researchers have suggested hybrid integration technology, which involves using a lower coating of high thermal conductivity silica to increase the response speed of polymer thermo-optic devices. Finally, compared with inorganic materials, polymers may exhibit a lower thermal stability under high temperatures and long working conditions. This may lead to the degradation of device performance or loss of functionality. Hence, stability at high temperatures is also something that researchers need to consider when selecting materials. Nevertheless, progress has been made in improving the thermal stability of polymers as optical waveguide materials. Polyimides, which are often used as optical waveguide materials, have the advantage of high thermal stability and can be used in high-temperature environments up to 400 °C without much damage to the mechanical properties [142]. In addition, most polymers are able to be modified and fluorinated to obtain higher thermal stability, degradation temperature, glass transition temperature, and mechanical properties [143].

## 6. Conclusions

This article reviewed the research progress of polymer devices based on the thermo-optic effect. Firstly, the principle of the thermo-optic effect was introduced. To facilitate a comparative analysis with polymer materials, we outlined the key properties and characteristics of various materials, including polymers, silicon, silicon dioxide, and silicon nitride. In terms of thermo-optic devices, subsequent to an introduction to the fundamental waveguide structures, we focused on three devices including thermo-optic switches, thermo-optic VOAs, and thermo-optic temperature sensors. Leveraging the high thermo-optic coefficient of polymeric materials, thermo-optic switches, and thermo-optic VOAs based on polymers offers significant advantages in terms of low power consumption. Moreover, the efforts made by researchers to reduce the power consumption and response time of the devices were reviewed. In the context of thermo-optic temperature sensors, the work to improve sensitivity from a material and structural perspective was reviewed. Furthermore, the flexibility of polymers allows for diverse fabrication methods for optical devices. We presented the several techniques for the preparation of polymer-based optical waveguides that can be utilized in the fabrication of thermo-optic devices. In conclusion, we addressed the challenges faced by polymer-based thermo-optic devices in the context of the demand for integrated and commercialized photonic devices. A comprehensive discussion and outlook were presented. The adjustability and flexibility inherent in polymer materials showcase tremendous potential for meeting diverse application requirements. With further research and development efforts dedicated to polymer materials, an excellent platform will be established for the integration and application of thermo-optic devices.

## Figures and Tables

**Table 1:** Related material parameters for the manufacture of thermo-optic devices.

Material	Refractive Index (@1550 nm)	TOC (K^−1^) ^1^	Thermal Conductivity (W/(m·K))	TEC (10^−6^ K^−1^) ^2^	$C_{P}$ (J/(kg·K)) ^3^
Silicon	3.45	1.86 × 10^−4^	163	2.6	700
Silica	1.45	0.62 × 10^−5^~1.28 × 10^−5^	1.4	0.5	730
Silicon nitride	2.1105	2.51 × 10^−5^	30	3	710
Polymer	1.3~1.7	−10^−3^~−10^−4^	0.1~0.3	10~220	1000~2000

^1^ TOC, thermo-optical coefficient; ^2^ TEC, thermal expansion coefficient; ^3^ $C_{P}$, heat capacity at a constant pressure.

**Table 3:** Performance comparison of the temperature sensors.

Material	Structure	Sensitivity (pm/°C)	Range (°C)	Reference
SOI	AMZI	438	16	[99]
	AMZI	445	40	[103]
	CMZI	1753.7	40	[104]
	CMRR	293.9	56.85	[94]
	MRR with Fano resonance	75.3	80	[105]
	MI	113.7	82	[106]
Silicon/Silicon Nitride	AMZI	324	-	[107]
SOI/TiO_2_	CMRR	64.34	20	[90]
Polymer	AMZI	30,800	2	[100]
	WBG	249	30	[101]
Polymer/Silica	AMZI	431	50	[108]
	MRR	228.6	12	[4]
	WBG	150	10	[102]
Polymer/Silicon	AMZI	172	7.7	[85]

## Data Availability

No new data were created or analyzed in this study. Data sharing is not applicable to this article.
